# A Comprehensive Protocol for Bayesian Phylogenetic Analysis Using MrBayes: From Sequence Alignment to Model Selection and Phylogenetic Inference

**DOI:** 10.21769/BioProtoc.5276

**Published:** 2025-04-20

**Authors:** Jinxing Wang, Fangmin Chen, Xu Xiao, Xinyao Yang, Wanting Xia

**Affiliations:** 1 Liaoning Province Key Laboratory of Urban Integrated Pest Management and Ecological Security, College of Life Science and Bioengineering, Shenyang University, Liaoning, China; 2 Key Laboratory of Ecological Restoration of Regional Contaminated Environment, Ministry of Education, Shenyang University, Shenyang, Liaoning, China; 3 School of Information Engineering, Wenzhou Business College, Wenzhou, Zhejiang, China

**Keywords:** Bayesian phylogenetic analysis, MrBayes, GUIDANCE2, MAFFT, ProtTest, MrModeltest, Sequence alignment, Evolutionary model selection

## Abstract

Bayesian phylogenetic analysis is essential for elucidating evolutionary relationships among organisms. Traditional methods often rely on fixed models and manual parameter settings, which can limit accuracy and efficiency. This protocol presents an integrated workflow that leverages GUIDANCE2 for rigorous sequence alignment, ProtTest and MrModeltest for robust model selection, and MrBayes for phylogenetic tree estimation through Bayesian inference. By automating key steps and providing detailed command-line instructions, this protocol enhances the reliability and reproducibility of phylogenetic studies.

Key features

• Robust sequence alignment: Combines GUIDANCE2 and MAFFT to handle complex evolutionary events.

• Automated model selection: Utilizes ProtTest and MrModeltest for protein evolution models and nucleotide substitution models, respectively.

• Streamlined workflow: Provides step-by-step instructions from sequence alignment to phylogenetic tree estimation through Bayesian inference.

## Background

Phylogenetic analysis plays a critical role in understanding the evolutionary relationships among species, informing diverse fields such as evolutionary biology, epidemiology, and conservation genetics. The process of generating a phylogenetic tree typically involves key steps including sequence alignment, model selection, and tree inference, each of which is essential for deriving reliable evolutionary conclusions. However, traditional phylogenetic workflows often involve manual sequence alignment and model selection, introducing potential biases and inefficiencies.

To address these challenges, numerous computational tools have been developed. For example, GUIDANCE2 enhances sequence alignment by accounting for alignment uncertainty and evolutionary events such as insertions and deletions [1]. Model selection tools like Protest [2] and MrModeltest2 [3] automate the identification of optimal evolutionary models using statistical criteria such as AIC and BIC, thereby improving the reliability of downstream phylogenetic inferences. Besides, tools such as PAUP* [4] enable comprehensive phylogenetic analysis for nucleotide sequences, while MEGA X [5] facilitates sequence format conversion and preliminary analyses.

Beyond these tools, several non-Bayesian phylogenetic inference methods offer powerful alternatives with distinct advantages. The PHYLIP package [6] provides a comprehensive suite of programs implementing distance matrix, maximum parsimony, and maximum likelihood methods, making it a versatile choice for diverse phylogenetic analyses. Maximum likelihood-based programs like RAxML [7] and IQ-TREE [8] have revolutionized the field with their computational efficiency and accuracy, especially for large datasets. FastTree [9] employs heuristic approaches to construct approximately maximum-likelihood phylogenetic trees with remarkable speed while maintaining reasonable accuracy. PhyML [10] offers robust algorithms for maximum likelihood tree estimation with extensive substitution model options and branch support assessment. These non-Bayesian tools provide complementary strengths to Bayesian methods, often excelling in computational efficiency while still delivering statistically sound phylogenetic inferences.

Bayesian methods, particularly those implemented in MrBayes [11], provide a robust probabilistic framework for estimating phylogenetic trees and evolutionary parameters by incorporating uncertainty and prior knowledge. However, integrating these tools into a cohesive and reproducible workflow remains challenging due to differing format requirements between tools. For example, GUIDANCE2 accepts FASTA/PHYLIP inputs, MrBayes requires NEXUS format [12], and PAUP* demands non-interleaved NEXUS [4,13] for its analyses. These diverging specifications create hidden technical barriers. Our protocol addresses these challenges by presenting a seamless, step-by-step guide that integrates sequence alignment, model selection, and Bayesian inference using MrBayes. It automates critical steps, minimizes manual intervention, reduces potential errors, and ensures reproducibility. Custom Python scripts are included to streamline the parsing of model selection outputs, enhancing data handling efficiency. This structured protocol simplifies the phylogenetic analysis process, improves the accuracy and reliability of results, and is applicable to diverse datasets, including both protein and nucleotide sequences.

The NEXUS format is a common data format for phylogenetic analysis, facilitating greater cooperation in the analysis and visualization of data. PAUP* reads data in NEXUS file format, and all NEXUS files must begin with the declaration "#NEXUS". The Newick format [14] is another widely used format for representing phylogenetic trees, and it is supported by many phylogenetic analysis tools. The protocol leverages MEGA for initial format conversions and PAUP* for format refinement, ensuring seamless data handoffs between tools and preventing pipeline failures from format mismatches. This approach systematically addresses integration challenges and provides a versatile and reliable resource for researchers conducting rigorous evolutionary studies.

## Software and datasets

All procedures in this protocol were developed and tested on Windows 10.

1. Python (Version: 3.13.1)

a. Homepage: https://www.python.org/

b. Downloads: https://www.python.org/ftp/python/3.13.1/python-3.13.1-amd64.exe

c. Platform: Windows

d. Last accessed: February 2025

e. Installation steps: Follow the instructions in the software installation package to install.

2. JAVA (Version: 8 or later)

a. Homepage: https://www.java.com/en/

b. Downloads: https://www.java.com/en/download/

c. Platform: Windows

d. Last accessed: February 2025

e. Installation steps: Follow the instructions in the software installation package to install.

3. PAUP* (Version: 4.0a Build 169)

a. Homepage: https://paup.phylosolutions.com/

b. Downloads: https://phylosolutions.com/paup-test/

c. Platform: Windows

d. Last accessed: February 2025

e. Installation steps: Follow the instructions in the software installation package to install.

4. MEGA X

a. Homepage: https://www.megasoftware.net/

b. Downloads: https://www.megasoftware.net/dload_win_gui

c. Platform: Windows

d. Last accessed: February 2025

e. Installation steps: Follow the instructions in the software installation package to install.

5. MrModeltest2 (Version: 2.4)

a. Homepage: https://github.com/nylander/MrModeltest2

b. Downloads: https://github.com/nylander/MrModeltest2/releases/tag/v.2.4

c. Platform: Windows, dependent on PAUP*

d. Last accessed: February 2025

e. No software installation is required: Copy the MrModelblock file from MrModelTest to your working directory, execute it in PAUP* via *File* > *Execute*, and use the generated mrmodel.scores file for subsequent analyses.

6. ProtTest (Version: 3.4.2)

a. Homepage: https://github.com/ddarriba/prottest3

b. Downloads: https://github.com/ddarriba/prottest3/releases/tag/3.4.2-release

c. Platform: Windows, Dependent on JAVA

d. Last accessed: February 2025

e. Installation steps: Download the latest ProtTest version (prottest-3.4.2-20160508.tar.gz) from its GitHub page and ensure Java is already installed. Extract the files to a directory with only English characters and no spaces. To use ProtTest, navigate to its extraction path in the command line terminal.

7. MrBayes (Version: 3.2.7a)

a. Homepage: https://nbisweden.github.io/MrBayes/

b. Downloads: https://github.com/NBISweden/MrBayes/releases/tag/v3.2.7

c. Platform: Windows

d. Last accessed: February 2025

e. Installation steps: Download MrBayes-3.2.7-WIN.zip from its GitHub page and extract it to a directory with only English characters and no spaces; inside the extracted bin folder, rename mb.3.2.7-win64.exe (for 64-bit CPUs) or mb.3.2.7-win32.exe (for 32-bit CPUs or if unsure) to mb.exe; place your NEXUS files in this directory, open a command line terminal here by holding the Shift key, right-clicking inside the folder, and selecting Open command window here (or Open PowerShell window here), then type mb and press Enter to launch MrBayes.

**System requirements**

To enhance the reproducibility and accessibility of this protocol, the following hardware specifications are recommended as minimal requirements for computational implementation:

1. Processor: A single-core central processing unit (CPU) with a base clock speed ≥2.0 GHz

2. Memory: 2 GB of random access memory (RAM)

3. Storage: 15 GB available disk space for software installations, intermediate files, and output storage

4. Graphics: No graphical processing unit (GPU) acceleration required

While these specifications suffice for basic analyses, multi-core processors (>4 cores) and expanded RAM (≥8 GB) are strongly recommended for improving computational efficiency during Bayesian inference with large datasets.

## Procedure

**Figure 1.**
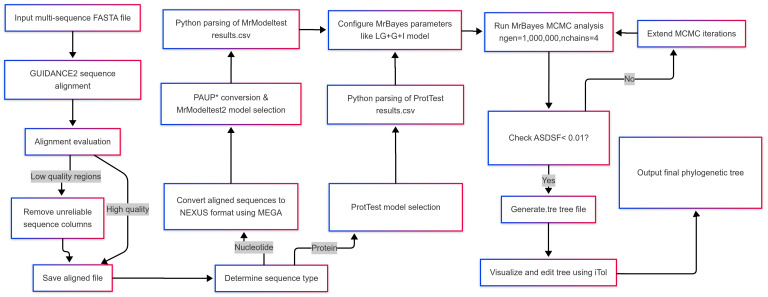
Integrated workflow for Bayesian phylogenetic analysis applying automated model selection

This protocol provides a systematic workflow for Bayesian phylogenetic analysis, integrating automated tools to enhance accuracy and reproducibility. The procedure comprises five main stages: (1) robust sequence alignment using GUIDANCE2 with MAFFT to handle evolutionary complexities; (2) sequence format conversion via MEGA and PAUP* for downstream compatibility; (3) optimal evolutionary model selection via ProtTest (proteins) or MrModeltest (nucleotides) guided by statistical criteria (AIC/BIC); (4) execution of Bayesian inference in MrBayes under the selected model parameters, including MCMC diagnostics; and (5) validation and visualization of phylogenetic outputs. These stages form a cohesive pipeline (Figure 1), minimizing manual intervention while ensuring biological rigor. Below, we detail each step with explicit command-line instructions, troubleshooting annotations, and Python scripts to streamline analysis.

**A. Sequence alignment**

Perform sequence alignment using GUIDANCE2, selecting MAFFT as the alignment tool.

**Figure 2.**
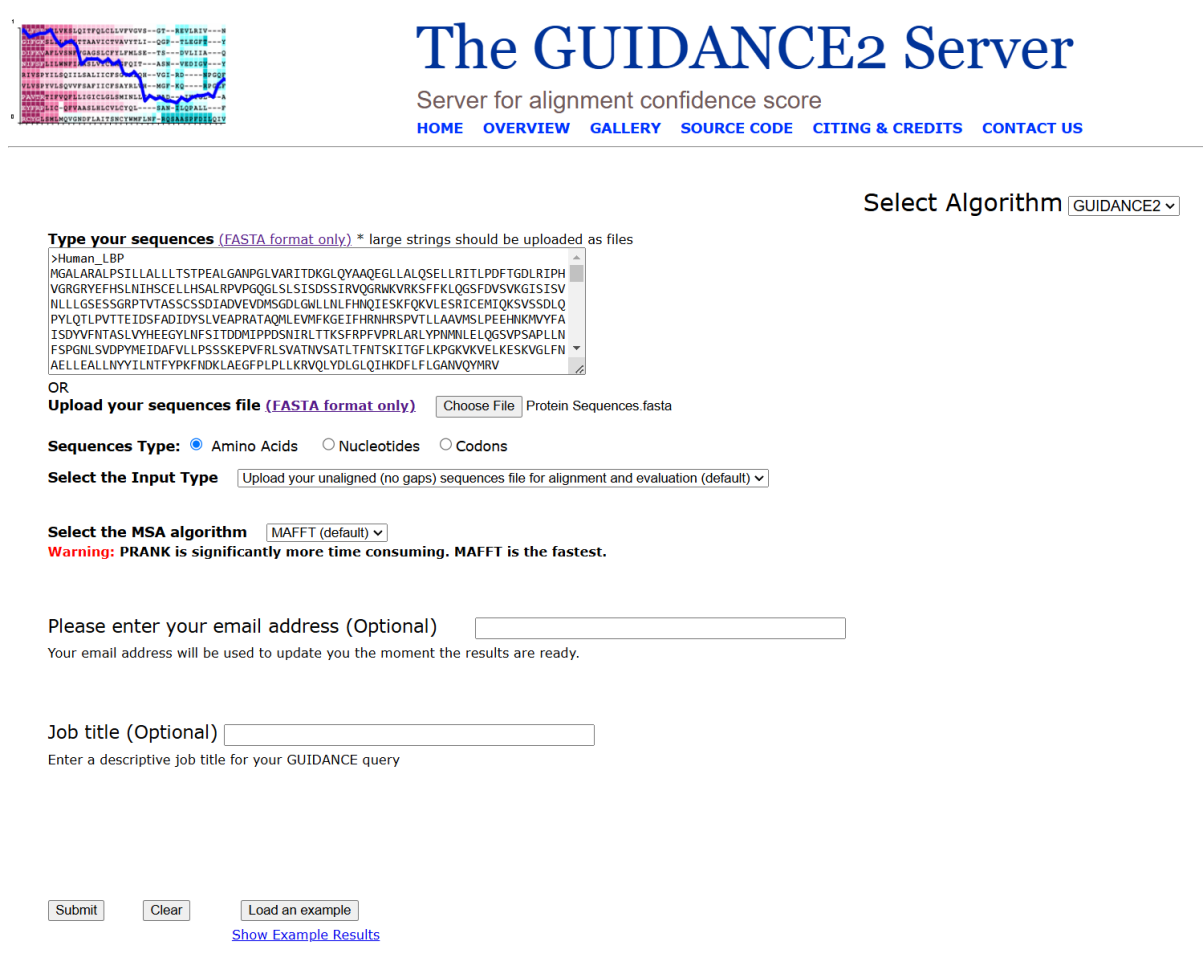
Basic operation of the GUIDANCE2 website

1. Access the GUIDANCE2 Server.

2. Upload sequence file. Upload your multi-sequence FASTA file to GUIDANCE2. Ensure the file format is correct (FASTA requires a header line starting with “>” followed by sequence lines; see FASTA format specifications for details) and that sequence names do not contain special characters (only numbers, letters, and underscores “_” are allowed).

3. Select the *alignment* tool. In the alignment tool options, choose *MAFFT* as the alignment method. The GUIDANCE2 web interface, including sequence input fields and alignment configuration options, is illustrated in Figure 2.

4. Configure alignment parameters. Users can adjust the advanced MAFFT options located at the bottom of the GUIDANCE2 webpage according to their specific needs.

*Note: Default MAFFT parameters are recommended for most datasets. However, parameter adjustments may enhance alignment accuracy for specialized cases, as follows:*

*a. For datasets with high complexity, consider using the Max-Iterate option (0, 1, 2, 5, 10, 20, 50, 80, 100, 1,000) in GUIDANCE2 to optimize alignment iterations.*

*b. When selecting a pairwise alignment method, it is crucial to consider the specific characteristics of the sequences and the objectives of the analysis. The 6mer method is suitable for shorter sequences or rapid preliminary analyses, utilizing short segments of six nucleotides for alignment, which offers speed but may compromise accuracy with complex sequences. The localpair approach is ideal for sequences with local similarities or conserved regions, effectively handling insertions and deletions (indels) and accommodating significant variability. In contrast, genafpair is best for longer sequences or when a global alignment is required, as it performs comprehensive alignments that can manage larger variations, making it particularly useful in phylogenetic analyses. The globalpair method is designed for global alignments, especially when sequences are of similar length, providing a holistic comparison. To choose the appropriate method, consider the sequence length—using 6mer or localpair for shorter sequences and genafpair or globalpair for longer ones—along with the degree of sequence similarity and the specific research objectives. By evaluating these factors, researchers can effectively select the most suitable pairwise alignment method for their analyses.*

**Figure 3.**
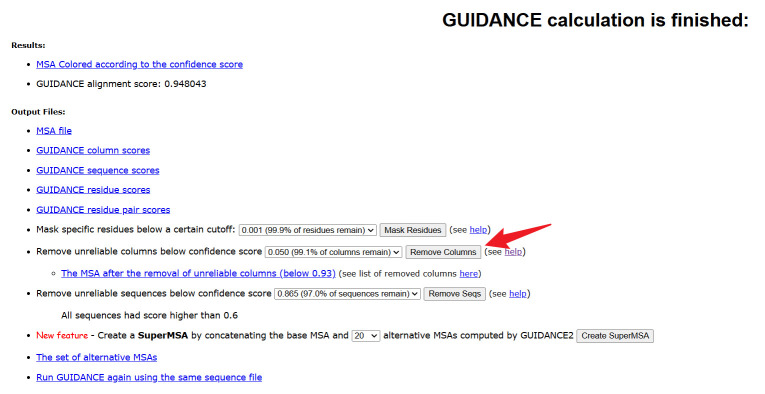
Remove unreliable columns operation

5. Run alignment. Click the *Submit* button and wait for the process to complete. Once finished, download the alignment result file (in FASTA format).

6. Evaluate alignment results. Perform an initial review of the alignment to ensure that sequences are properly aligned.

7. Remove unreliable alignment columns. On the GUIDANCE2 website, identify and exclude unreliable alignment columns based on the preliminary experiments. Ensure that the final alignment is of high quality and biologically meaningful (Figure 3).

*Notes:*

*1. MAFFT is used in this protocol due to its strong performance; however, you may also consider PRANK, which explicitly models insertions and deletions as evolutionary events and can be advantageous for highly complex sequences. Note that its accuracy may occasionally be lower than MAFFT’s. We recommend conducting preliminary tests—using different alignment tools such as PRANK or ClustalW, followed by phylogenetic tree construction—to determine which approach best suits your data. Comparing these results will help you select the most appropriate alignment method.*

*2. For users with advanced expertise in sequence comparison, the MEGA program may be utilized to conduct sequence alignment, followed by manual elimination of unreliable columns.*

**Caution:** Ensure that the uploaded sequence files are correctly formatted to prevent alignment failures or inaccurate results.

**B. Sequence format conversion**

Both PAUP* and MrBayes require sequence data in NEXUS format (.nex).

**Figure 4.**
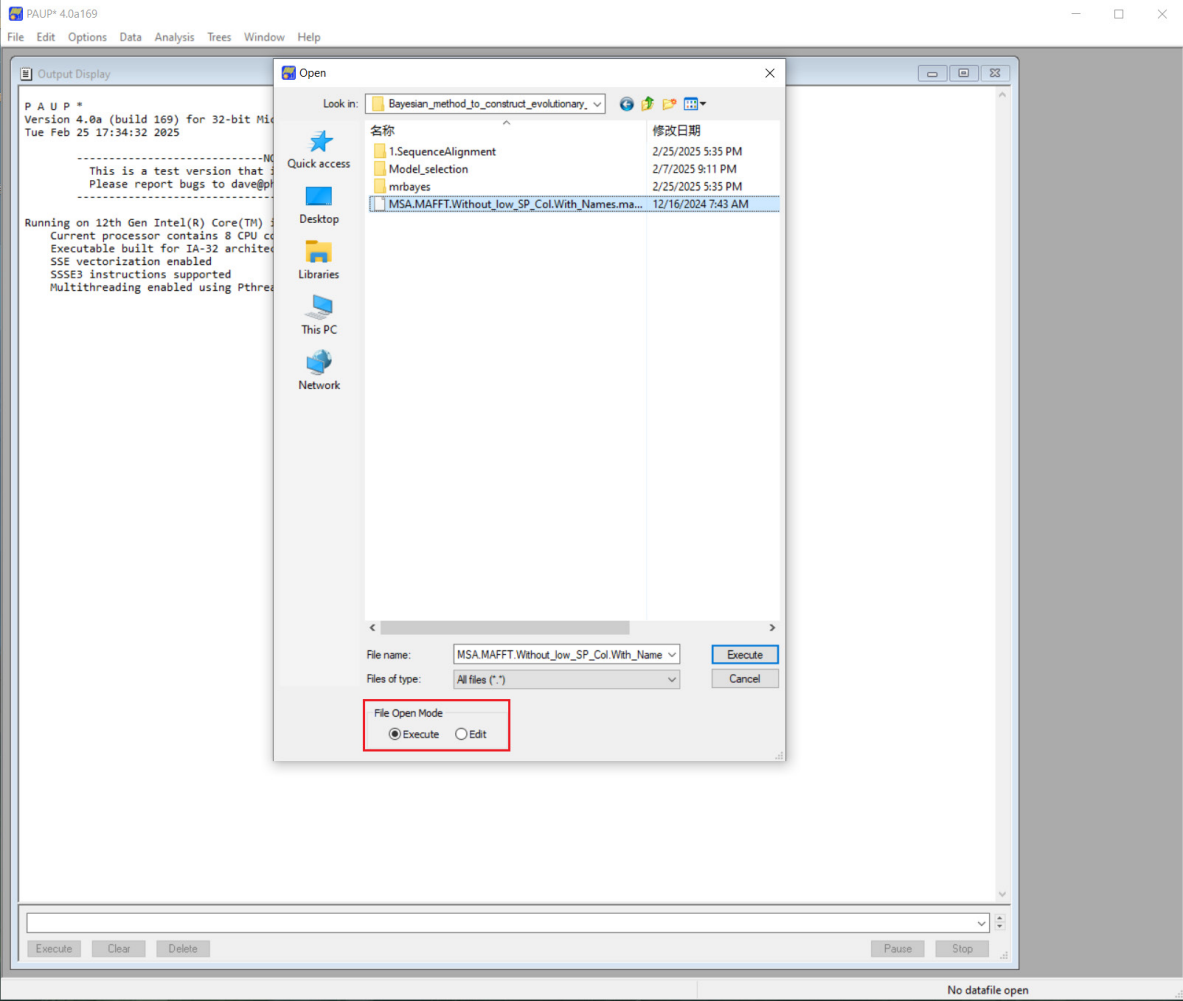
Import a NEXUS file using the *Execute* mode

**Figure 5.**
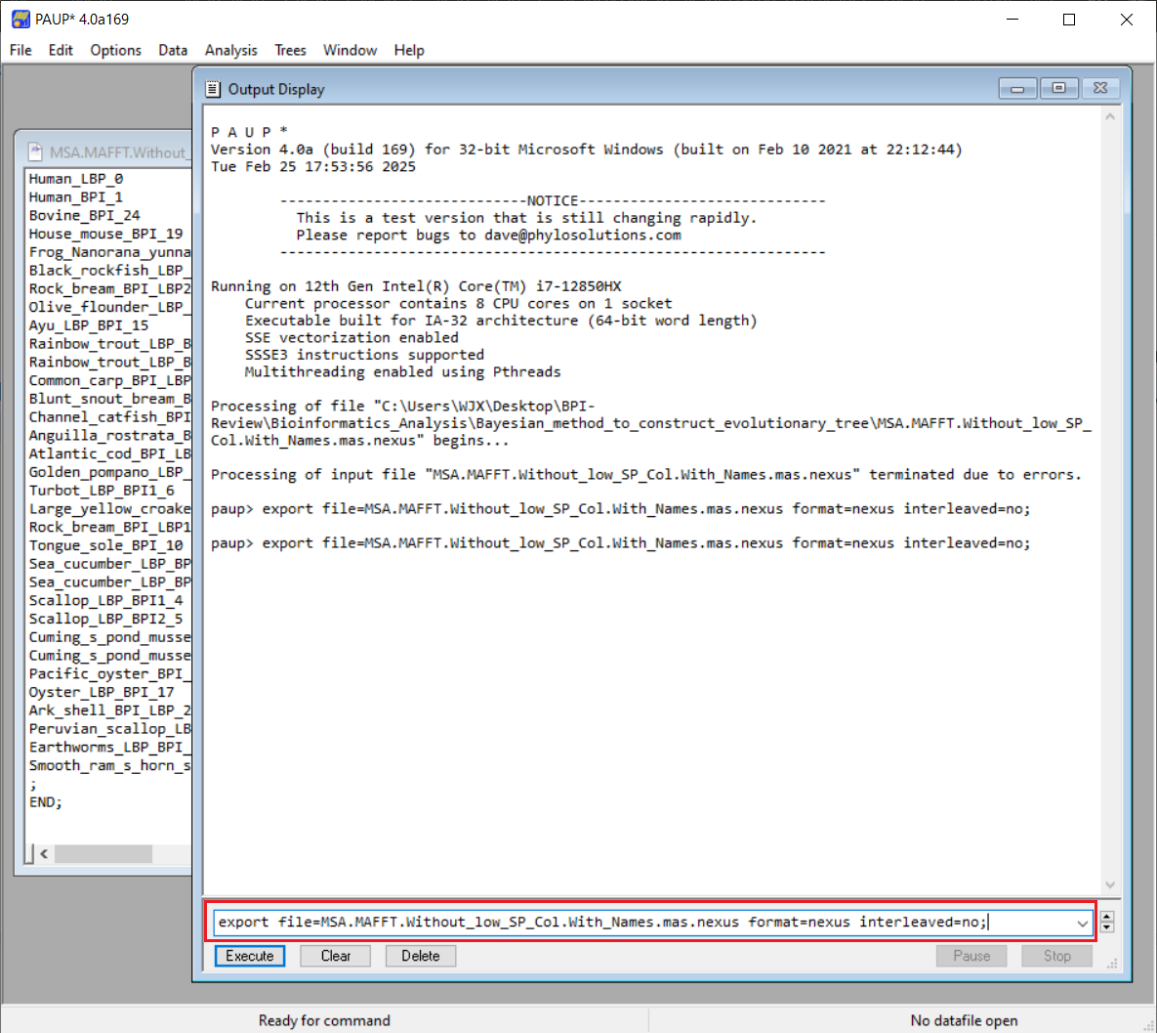
Convert interleaved format sequences to non-interleaved format sequences using PAUP*

*Note: Verify that all sequence names contain no illegal characters. Specifically, only letters, numbers, and underscores are supported.*

1. Convert FASTA to NEXUS using MEGA. Prepare alignment: Ensure your multiple sequence alignment is in FASTA format. Open MEGA and import the FASTA file. Export the alignment as a NEXUS file.

2. To convert interleaved to non-interleaved NEXUS for nucleotide datasets, launch the PAUP software. Go to the *File* menu, click *Open*, and select the NEXUS file exported from MEGA using the *Execute* option, as shown in Figure 4. **Skip this step** if using ProtTest for protein sequence model selection.

3. In the PAUP command line (displayed in Figure 5), type the following command and execute it:

export file=filename.nex format=nexus interleaved=no

Replace filename.nex with your desired output filename (typically the same as the original). After entering the command, click the *Execute* button to run.

**C. Protein model selection: ProtTest**

Section C is applicable to protein sequences. If working with nucleic acid sequences, the user should proceed directly to Section D.

1. Data preparation. Ensure your multiple sequence alignment is saved in FASTA or PHYLIP format.

2. Download and install ProtTest. Obtain the latest version of ProtTest from its GitHub repository. ProtTest requires Java to run. If not already installed, download and install Java from the official website.

3. Run ProtTest. Open a command-line terminal and navigate to the ProtTest installation directory. Execute the following command, replacing <input_file> and <output_file> with your specific filenames:

java -jar prottest-3.4.2.jar -i <input_file> -o <output_file> -all-distributions -F -AIC -BIC -tc 0.5 -threads 12

Parameters explanation:

-i <input_file>: Specifies the input alignment file.

-o <output_file>: Defines the output file for results.

-all-distributions: Tests all possible substitution model distributions.

-F: Enables the empirical base frequencies.

-AIC -BIC: Calculates Akaike information criterion and Bayesian information criterion scores.

-tc 0.5: Sets the transition/transversion ratio.

-threads 12: Utilizes 12 CPU threads for faster computation, decided by the user's own computer configuration.

4. Parse ProtTest output. To facilitate easier analysis, you can use the following Python script to extract model information and save it as a CSV file.

"""

Script Functionality:

---------------------

Extracts model names, AIC, and BIC scores from ProtTest output and saves them as a CSV file.

Dependencies:

1. pandas

 - For handling tabular data.

 - Installation: pip install pandas

2. tqdm

 - For displaying progress bars.

 - Installation: pip install tqdm

Usage:

------

1. Update `file_path` with the path to your ProtTest output file.

2. Update `output_path` with the desired path for the CSV output.

3. Run the script:

 python parse_prottest_output.py

"""

import pandas as pd

from tqdm import tqdm

# User-defined paths

file_path = r"<YOUR_INPUT_FILE_PATH>" # ProtTest output file

output_path = r"<YOUR_OUTPUT_FILE_PATH>" # Desired CSV output path

# Initialize lists to store data

models = []

aic_values = []

bic_values = []

# Count total lines for progress bar

try:

 with open(file_path, "r") as file:

 total_lines = sum(1 for line in file)

except Exception as e:

 print(f"Error counting lines: {e}")

 total_lines = 0

# Parse ProtTest output

try:

 with open(file_path, "r") as file:

 for line in tqdm(file, total=total_lines, desc="Processing lines"):

 if not line.strip() or line.startswith("#"):

 continue

 if line.startswith("Model") or not line[0].isalpha():

 continue

 columns = line.split()

 if len(columns) >= 5:

 try:

 model = columns[0]

 aic = float(columns[2])

 bic = float(columns[4])

 models.append(model)

 aic_values.append(aic)

 bic_values.append(bic)

 except ValueError:

 print(f"Skipping invalid line: {line.strip()}")

except Exception as e:

 print(f"Error reading file: {e}")

# Verify data consistency

if len(models) == len(aic_values) == len(bic_values):

 df = pd.DataFrame({

 "Model": models,

 "AIC": aic_values,

 "BIC": bic_values

 })

 df.to_csv(output_path, index=False)

 print(f"Data successfully saved to {output_path}")

else:

 print("Error: Inconsistent data lengths. Please check the parsing logic.")

Instructions:

Ensure Python is installed on your system and install the required libraries:

pip install pandas tqdm

Update the file_path and output_path variables in the script with your specific file paths. Save the script as parse_prottest_output.py and run it:

python parse_prottest_output.py

The script will generate a CSV file containing model names along with their corresponding AIC and BIC scores.

5. Select the best-fit model:

Review the ProtTest output or the generated CSV file. Identify the model with the lowest AIC or BIC score. This model is recommended for subsequent phylogenetic analyses to ensure reliable results.

**D. Nucleotide model selection: MrModeltest**

1. Prepare the NEXUS file. Ensure your nucleotide sequence alignment is saved in non-interleaved NEXUS format (.nex).

2. Run MrModeltest. Open PAUP and load your NEXUS file. In PAUP, open and select the MrModelblock file found in the MrModeltest2-v.2.4/doc/ directory within the extracted archive. This will generate a *mrmodel.scores* file in your current directory. Navigate to the directory containing mrmodel.scores from the command line. Execute the following command to perform model selection:

mrmodeltest2 < mrmodel.scores > out.txt

This command processes the scores and outputs the results to out.txt. You can rename out.txt as desired.

3. Parse MrModeltest output. Use the following Python script to extract and organize model selection results into a CSV file.

import re

import csv

import chardet

# Function to detect the encoding of a file

def detect_encoding(file_path):

    """Detect the encoding of the input file."""

    with open(file_path, 'rb') as file:

        raw_data = file.read()

    result = chardet.detect(raw_data)

    return result['encoding']

# Function to read the MrModeltest output file

def read_results(file_path):

    """Read file content using the detected encoding."""

    detected_encoding = detect_encoding(file_path)

    print(f"Detected file encoding: {detected_encoding}")

    with open(file_path, 'r', encoding=detected_encoding, errors='ignore') as file:

        return file.read()

# Function to extract models from the MrModeltest output

def extract_all_models(content):

    """Extract all model information from MrModeltest output."""

    models = []

    # Extract data from the AIC weights table

    # Use a more specific pattern to match the table rows

    table_pattern = r"Model\s+-lnL\s+K\s+AIC\s+delta\s+Weight\s+CumWeight\s*\n[-\s]+\n(.*?)[-]{5,}"

    table_match = re.search(table_pattern, content, re.DOTALL)

    if table_match:

        print("AIC weights table found")

        table_content = table_match.group(1)

        # Process each line

        for line in table_content.strip().split('\n'):

            if not line.strip():

                continue

            # Print the line for debugging

            print(f"Table line: {line}")

            # Try to extract model data using a specific pattern

            model_match = re.match(r"(\w+\+?\w*)\s+(\d+\.\d+)\s+(\d+)\s+(\d+\.\d+)\s+(\d+\.\d+)\s+([0-9.e-]+)", line)

            if model_match:

                model_name = model_match.group(1)

                lnl = float(model_match.group(2))

                k = int(model_match.group(3))

                aic = float(model_match.group(4))

                delta = float(model_match.group(5))

                weight = float(model_match.group(6))

                models.append({

                    'Model': model_name,

                    'lnL': lnl,

                    'K': k,

                    'AIC': aic,

                    'Delta': delta,

                    'Weight': weight,

                    'Method': 'AIC table'

                })

    # If no models found via table, extract the best models

    if not models:

        print("No models found in table, extracting best models")

        # Extract hLRT selected model

        hLRT_match = re.search(r"Model selected:\s+(\w+\+\w+).*?-lnL\s+=\s+(\d+\.\d+)", content, re.DOTALL)

        if hLRT_match:

            models.append({

                'Model': hLRT_match.group(1),

                'lnL': float(hLRT_match.group(2)),

                'Method': 'hLRT',

                'AIC': None,  # Add all possible fields with default values

                'K': None,

                'Delta': None,

                'Weight': None

            })

        # Extract AIC selected model

        aic_match = re.search(r"Model selected:\s+(\w+\+\w+).*?-lnL\s+=\s+(\d+\.\d+).*?AIC\s+=\s+(\d+\.\d+)", content, re.DOTALL)

        if aic_match:

            models.append({

                'Model': aic_match.group(1),

                'lnL': float(aic_match.group(2)),

                'AIC': float(aic_match.group(3)),

                'Method': 'AIC selected',

                'K': None,

                'Delta': None,

                'Weight': None

            })

    # Try direct extraction of all model lines as last resort

    if len(models) <= 2:

        print("Trying direct extraction of model lines")

        # Look for lines that match model patterns

        all_lines = content.split('\n')

        for line in all_lines:

            # Skip empty lines or header lines

            if not line.strip() or 'Model' in line or '-' * 5 in line:

                continue

            # Extract models with a more flexible pattern

            model_match = re.match(r"^(\w+\+?\w*)\s+(\d+\.\d+)\s+(\d+)\s+(\d+\.\d+)", line.strip())

            if model_match:

                print(f"Found model line: {line}")

                model_parts = re.split(r'\s+', line.strip())

                if len(model_parts) >= 4:

                    model_data = {

                        'Model': model_parts[0],

                        'Method': 'Table extraction'

                    }

                    # Try to add all available fields

                    try:

                        if len(model_parts) > 1:

                            model_data['lnL'] = float(model_parts[1])

                        if len(model_parts) > 2:

                            model_data['K'] = int(model_parts[2])

                        if len(model_parts) > 3:

                            model_data['AIC'] = float(model_parts[3])

                        if len(model_parts) > 4:

                            model_data['Delta'] = float(model_parts[4])

                        if len(model_parts) > 5:

                            model_data['Weight'] = float(model_parts[5])

                        models.append(model_data)

                    except (ValueError, IndexError) as e:

                        print(f"Error parsing model line: {e}")

    print(f"Total models extracted: {len(models)}")

    return models

# Function to write all models to CSV file

def write_all_models_to_csv(file_path, output_csv):

    """Extract model information and write to CSV file."""

    content = read_results(file_path)

    # Save raw content for debugging

    with open(output_csv + '.debug.txt', 'w', encoding='utf-8') as debug_file:

        debug_file.write(content)

    models = extract_all_models(content)

    if not models:

        print("No models were found in the file.")

        return

    # Collect all unique fields from all models

    all_fields = set()

    for model in models:

        all_fields.update(model.keys())

    fieldnames = sorted(list(all_fields))

    print(f"Using fields: {fieldnames}")

    with open(output_csv, 'w', newline='') as csvfile:

        writer = csv.DictWriter(csvfile, fieldnames=fieldnames)

        writer.writeheader()

        for model in models:

            # Ensure all fields exist in each model

            for field in fieldnames:

                if field not in model:

                    model[field] = None

            writer.writerow(model)

    print(f"Successfully extracted {len(models)} models to {output_csv}")

# File paths

file_path = "out.txt"            # Replace with your MrModeltest output file path

output_csv = "model_results.csv" # Desired CSV output path

# Execute the script

write_all_models_to_csv(file_path, output_csv)

Instructions:

Save the script as parse_mrmodeltest_output.py. Update the file_path and output_csv variables with your specific file paths.

4. Run the script:

python parse_mrmodeltest_output.py

The script will generate a CSV file (model_results.csv) containing the model names, lnL values, and corresponding parameters.

5. Select the best-fit model:

Open the out.txt or the generated CSV file. Identify the model with the highest lnL (log-likelihood) value, which typically indicates the best-fit model for your nucleotide data. Use this model for subsequent phylogenetic tree construction to ensure accurate and reliable results.

*Notes:*

*1. Modify the file paths in the Python scripts to match your directory structure.*

*2. Ensure that input files are correctly formatted to prevent parsing errors.*

By following these optimized steps, you can efficiently select the most appropriate substitution models for both nucleotide and protein sequences, thereby enhancing the accuracy of your phylogenetic analyses.

**E. Running MrBayes with the optimal model**

1. Place your NEXUS file in the same directory as mb.exe. Open a command line here by navigating to the directory (e.g., cd C:\path\to\folder) **or** using *Shift+Right-Click* > *Open command* window, then launch MrBayes by typing *mb* and pressing *Enter*.

*Note: If using PowerShell, prefix the command with ./ (e.g., ./mb) to execute the program.*

2. Load the NEXUS file. At the MrBayes prompt, load your NEXUS file by executing:

execute your_file_name.nexus

3. To avoid compatibility issues, disable the use of the Beagle library (optional) by entering:

set usebeagle=no

4. Access your model and parameter settings. Open the Word file containing the model and parameter settings in Table S1 or Table S2. Identify the desired model (e.g., LG, WAG, jones) and parameter combination (e.g., I+G+F, G+F) from the table. Each combination corresponds to specific prset and lset commands.

5. Copy and paste the commands into MrBayes. For example, users can select LG model with the I+G+F parameter combination:

prset aamodelpr = fixed(LG);

prset statefreqpr = fixed(empirical);

lset rates = invgamma;

6. After setting the model and parameters, start the Markov chain Monte Carlo (MCMC) analysis by entering:

mcmc ngen=1000000 samplefreq=100 printfreq=1000 diagnfreq=1000 nchains=4;

Command breakdown:

ngen=1000000: Number of generations (e.g., 1,000,000). It can be modified according to needs.

samplefreq=100: Frequency of sampling the chain (every 100 generations). Not recommended to modify.

printfreq=1000: Frequency of printing the log to the screen (every 1,000 generations). It has no effect on the results.

diagnfreq=1000: Frequency of diagnostic output (every 1,000 generations). No impact on the results.

nchains=4: Number of parallel chains (commonly set to 4).

*Note: The number of generations (ngen = 1,000,000) can be adjusted according to the user’s requirements. For instance, if convergence metrics (e.g., ASDSF) indicate inadequate sampling or instability, users may increase the ngen value to extend the MCMC run. The sampling frequency (samplefreq = 100) determines how often the chain is sampled; while smaller values improve effective sample size (ESS), frequent sampling may unnecessarily inflate file sizes. We recommend retaining the default settings unless explicitly required. Parameters printfreq = 1,000 and diagnfreq = 1,000 solely control the frequency of progress updates and diagnostic outputs on the screen, respectively, and do not impact the computational results. The number of parallel chains (nchains = 4) balances the exploration of parameter space and computational efficiency, where a higher number of chains facilitates improved convergence diagnostics while increasing resource demands. Users should verify convergence metrics (e.g., ASDSF < 0.01) before terminating runs and adjust parameters accordingly.*

7. Monitor the analysis. MrBayes will display progress logs, including current generation, likelihood, and diagnostic statistics. Monitor convergence by checking the average standard deviation of split frequencies (ASDSF) and consider extending run times if ASDSF > 0.01.

8. Extract and summarize the results. After completing the MCMC run, summarize the tree topology and parameter estimates by entering:

sumt burnin=250;

This command discards the first 250 generations as burn-in. Next, use the following code to obtain a summary of the parameter estimates:

sump burnin=250;

This provides statistics like mean, standard deviation, and confidence intervals for model parameters. To export the posterior trees for visualization, use:

sumptrees filename=your_tree.t burnin=250;

Replace your_tree.t with your desired filename.

**Figure 6.**
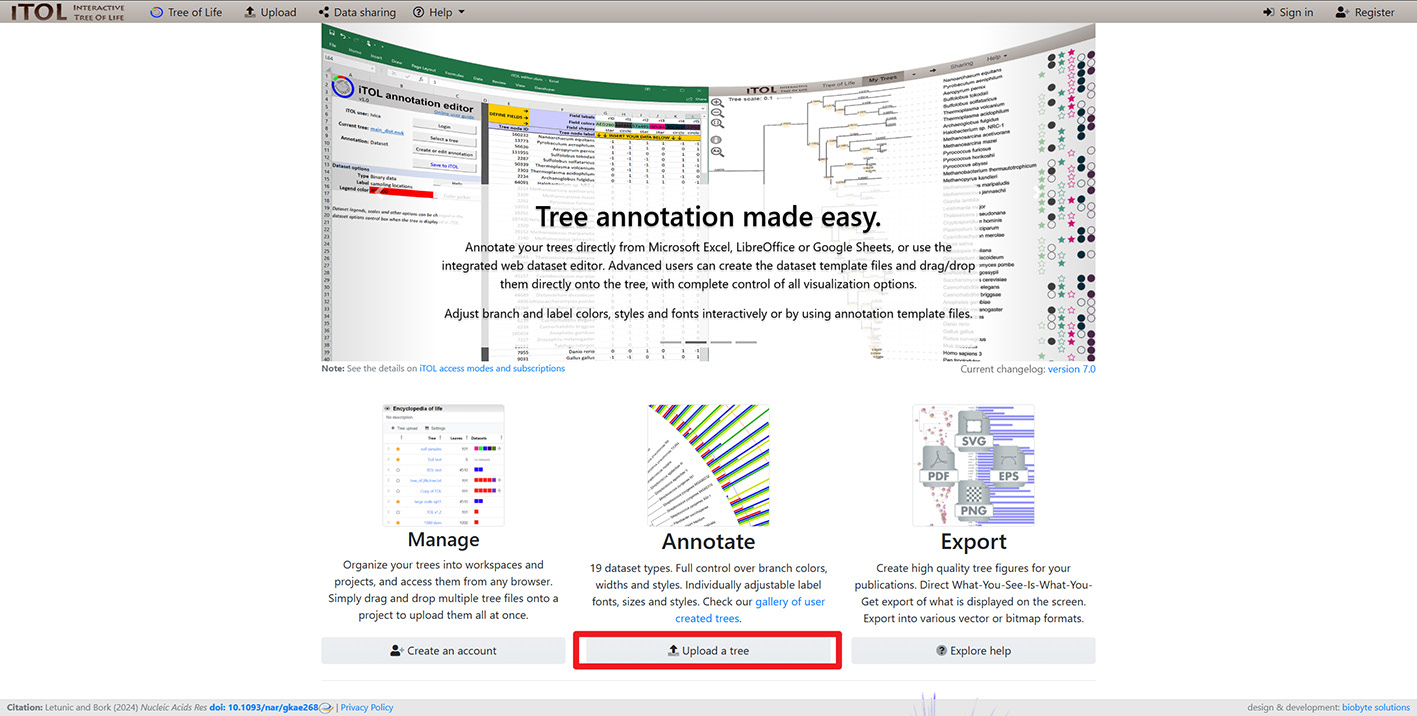
The iTol website

9. After the run is complete, the .*tre* file is the phylogenetic tree file generated from this run. This file cannot be opened with MEGA, so it is recommended to use the iTol website for viewing and editing. The interface of the iTol website is shown in Figure 6, where users can quickly edit and visualize the generated evolutionary tree by clicking the *Upload a tree* button.

## Validation of protocol

To validate the proposed protocol, lipopolysaccharide-binding protein (LBP) nucleotide sequences and bactericidal/permeability-increasing (BPI) protein sequences from various species were selected as validation materials. The specific sequences are provided in the supplementary materials (Datasets S1 and S2). Using these datasets, phylogenetic trees were constructed using the Bayesian method, as shown in Figure 1. Specifically, Bayesian analyses utilized 1,000,000 MCMC iterations. Numbers above each branch represent the confidence values (posterior probability) of the current branch. The results demonstrated a high degree of reproducibility across all constructed trees, highlighting the robustness of the protocol in generating accurate and consistent phylogenetic analyses. This establishes the protocol as a reliable framework for evolutionary studies.

**Figure 7.**
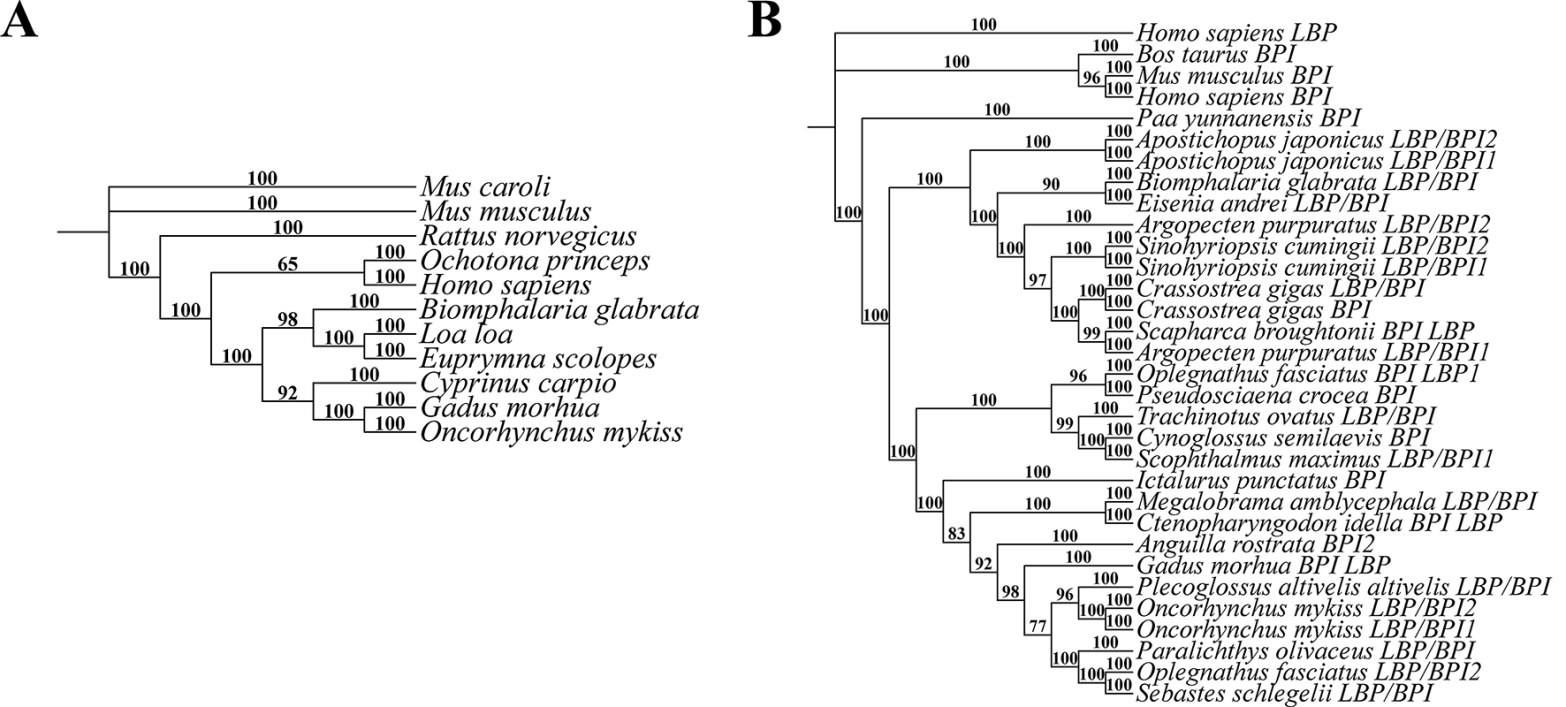
Phylogenetic trees constructed from lipopolysaccharide-binding protein (LBP) nucleotide sequences and BPI protein sequences across different species using the Bayesian method. (A) Phylogenetic tree of LBP nucleotide sequences [Markov chain Monte Carlo (MCMC) iterations: 1,000,000]. (B) Phylogenetic tree of BPI protein sequences (MCMC iterations: 2,000,000). Numbers above each branch represent the confidence values (posterior probability) of the current branch.

## General notes and troubleshooting

This section addresses common issues that may arise during Bayesian phylogenetic analysis and provides practical solutions to help users troubleshoot problems effectively.

1. Computational resources: While MrBayes can run on standard computers, analysis of large datasets (>100 taxa or >10,000 characters) may require extended run times. Consider starting with smaller ngen values (100,000) for initial tests before committing to longer runs.

2. File organization: Maintain a consistent directory structure throughout your analysis. Create separate folders for raw sequences, alignments, model selection outputs, and MrBayes results to prevent file management confusion.

3. Parameter files: Save command blocks for successful analyses as separate files that can be executed in future runs. This practice ensures reproducibility and saves time when running similar analyses.

4. In addition to the above, other general problems that users may encounter are shown in Table 1.

**Table 1.** Some common issues and corresponding solutions

Issue	Potential solution
GUIDANCE2 fails to process sequences	Check sequence names for special characters (spaces, slashes, colons); rename using only alphanumeric characters and underscores
Poor alignment quality	Try different alignment algorithms in GUIDANCE2 (e.g., PRANK)
Alignment appears to contain misaligned regions	Manually inspect alignment in MEGA or similar software; consider removing highly variable regions or using GUIDANCE2's column reliability scores to filter columns
MEGA fails to recognize FASTA format	Ensure sequence headers start with ">" and contain no illegal characters; check for hidden characters that may have been introduced during copying
"Invalid NEXUS format" error in PAUP*	Verify NEXUS file begins with "#NEXUS"; ensure all commands end with semicolons; check for interleaved vs. non-interleaved format mismatches
NEXUS file not recognized by MrBayes	Confirm file format meets MrBayes specifications; use PAUP* export function to ensure compatibility; verify character encoding (use UTF-8)
ProtTest fails with Java error	Verify Java 8+ is installed (not OpenJDK in some cases)
Model selection script fails to parse output	Check output file format; ensure version compatibility between tool output and parsing script; manually inspect file for unexpected format changes
Conflicting model selection between AIC and BIC	Prefer BIC for larger datasets and AIC for smaller ones; when in doubt, run analyses with both models and compare results
MrModeltest not producing output	Ensure PAUP* is properly installed and accessible; verify MrModelblock file is in the correct directory; check for proper file permissions
MrBayes fails to start	Verify executable path is correct; ensure the file is renamed properly (mb.exe); check system compatibility, and consider running the 32-bit version directly
MrBayes "Unexpected token" errors	Check command syntax carefully; ensure all commands end with semicolons; verify model parameters are compatible with the selected model
MCMC chains fail to converge	Increase ngen value (e.g., to 5–10 million); adjust heating parameter (temp = value); monitor average standard deviation of split frequencies (target <0.01)
Low posterior probabilities (<0.9)	Run a longer analysis; check model fit; consider revising the alignment; evaluate sequence quality and potential contamination
High memory usage or program crashes	Reduce number of chains; use checkpoint function (mcmcp autoclose = yes); partition data into smaller segments; run on more powerful hardware if available
Tree file (.tre) cannot be viewed	Use specialized software like FigTree or online viewers like iTOL (https://itol.embl.de/); verify file is not corrupted
Unexpected tree topology	Compare with analyses using different models; verify alignment quality; consider biological explanations for unexpected relationships
Parameter estimates have high variance	Increase burn-in value; run longer chains; check for proper mixing of chains in parameter trace files
Error opening tree file in visualization software	Convert tree file format using conversion tools (e.g., Newick utilities); check for format compatibility with visualization software

5. Advanced troubleshooting: For persistent issues, users are encouraged to consult the MrBayes manual for detailed explanations of parameters and error messages; search the ugene forum, where many common issues have been previously addressed; for alignment problems, refer to the GUIDANCE2 documentation for detailed parameter explanations; when encountering script execution issues, verify Python environment settings and package installations using pip list to confirm that all dependencies are correctly installed; and for model selection challenges, consult the original publications for ProtTest and MrModeltest to understand the statistical frameworks underlying different criteria.

Following these troubleshooting steps should resolve the majority of issues encountered during Bayesian phylogenetic analysis using this protocol. If problems persist, consider posting to specialized forums like Phylobabble or Biostars with detailed descriptions of the issues and steps already attempted.

**Validation strategy**

This protocol validation adopts a process-centric approach that prioritizes methodological reproducibility over phylogenetic accuracy assessment. Given that phylogenetic "truth" is inherently unknowable for empirical datasets, our validation strategy focuses on procedural consistency and statistical robustness rather than comparing topologies against a presumed ground truth. We selected LBP nucleotide sequences and BPI protein sequences as test cases due to their moderate complexity and documented evolutionary patterns.

The validation framework employs three complementary criteria: (1) workflow reproducibility, confirmed through independent replicate analyses yielding consistent tree topologies with high posterior probabilities (>0.95) for key evolutionary relationships; (2) computational stability, demonstrated by convergence diagnostics including average standard deviation of split frequencies consistently below 0.01 after 1,000,000 MCMC generations; and (3) statistical robustness, evidenced by the posterior probability values, which represent the Bayesian framework's native confidence metric and directly quantify the probability that a given clade is true given the data and model. Unlike bootstrap values in maximum likelihood approaches, these posterior probabilities inherently account for phylogenetic uncertainty and model parameter variation.

To facilitate step-by-step validation by users, we provide comprehensive supplementary materials that document each stage of the analytical pipeline:

1. Input datasets (Dataset_S1 and Dataset_S2): Complete BPI protein and LBP nucleotide sequence files in FASTA format, enabling direct replication of our alignment procedures.

2. Alignment quality assessment (File_S1): GUIDANCE2 alignment scores in HTML format, documenting column reliability and alignment confidence metrics used for filtering unreliable positions.

3. Model selection evidence (Files_S2 and S3): Complete outputs from MrModeltest and ProtTest analyses, allowing independent verification of model selection decisions for both nucleotide and protein datasets.

4. Parameter settings reference (Table_S1 and Table_S2): Comprehensive tables outlining the exact MrBayes command parameters for all commonly used evolutionary models, facilitating reproducible application across diverse datasets.

5. Analysis execution logs (File_S5): Full MrBayes run logs showing MCMC convergence diagnostics, parameter estimates, and computational performance metrics that validate our recommended settings.

By providing these materials, we enable complete pipeline validation by independent researchers at each analytical step, establishing a reproducible framework for diverse evolutionary studies while allowing users to adapt the protocol to their specific research questions.

## Supplementary information

The following supporting information can be downloaded here:

1. Dataset S1. The LBP sequences from different species

2. Dataset S2. The BPI sequences from different species

3. Table S1. Bayesian program model and parameter settings for protein sequences

4. Table S2. Bayesian program model and parameter settings for nucleic acid sequences

5. File S1. GUIDANCE2 scores

5. File S2. MrModeltest results

7. File S3. ProtTest results

8. File S4. MrBayes logs
